# Low Power and High Psychopathy: A Toxic Combination for Psychological Aggression

**DOI:** 10.1002/ab.70045

**Published:** 2025-08-06

**Authors:** Robert Körner, Astrid Schütz, Brad J. Bushman

**Affiliations:** ^1^ Department of Psychology Otto‐Friedrich‐University of Bamberg Bamberg Germany; ^2^ School of Communication The Ohio State University Columbus Ohio USA

**Keywords:** APIM, power, psychological aggression, psychopathy, relationships

## Abstract

Power and aggression are core relational variables that share a fickle relationship. It is unclear whether high or low power relates to psychological aggression and under which circumstances. We tested psychopathy as a potential moderator in the power‐aggression link because psychopathy is characterized by a lack of empathy and shallow emotional response. Psychopathy could strengthen the link between high power and psychological aggression because power ignites character traits and their corresponding behavior. Alternatively, psychopathy could strengthen the link between low power and psychological aggression because individuals high in psychopathy may attempt to compensate for their lack of power with aggression. We tested these competing hypotheses in a romantic context across two studies (*N*_1_ = 188 individuals, *N*_2_ = 226 couples). We found power to be negatively related to both actors' and partners' psychological aggression. Supporting the latter hypothesis, we found that the most psychologically aggressive people had low power and high psychopathy. In addition, people reported high psychological aggression when their *partners* were low in power and high in psychopathy. These findings advance existing power theories and research by highlighting how personality traits such as psychopathy affect both intra‐ and interpersonal links to psychological aggression.

Aggression is any behavior directed by a perpetrator toward a victim with the intent to cause harm, while the victim is motivated to avoid this behavior (Anderson and Bushman 2002). Psychological aggression means harming other people with words (e.g., yelling, swearing, name‐calling, threatening; Bushman and Huesmann 2010). In romantic relationships, the focus here, psychological aggression can mean behavior such as insulting, threatening, or accusing one's partner (Straus et al. 1996). Acts of psychological aggression are a widespread global phenomenon with severe health, social, and economic costs (World Health Organization 2022). Several factors may contribute to psychological aggression, including social power (Weick 2020). In fact, power has often been discussed as strongly intertwined with aggression (e.g., Overall et al. 2016; Ronfeldt et al. 1998).

However, the direction of the link between power and psychological aggression is unclear (e.g., Babcock et al. 1993; Cross et al. 2019; Ronfeldt et al. 1998), which is why initial studies reported moderators to be relevant (e.g., Fast and Chen 2009). Nevertheless, it is still poorly understood *when* power is associated with aggression, making further thorough tests of potentially moderating factors important to address. Additionally, the power‐aggression link is often studied at an individual level only, ignoring the fundamental interpersonal nature of power. By definition, power relationships involve two people—an actor and a partner (Anderson et al. 2012; Körner and Schütz 2021; Overall et al. 2023). Consequently, studying power and aggression on a dyadic level is necessary to understand their relationship more fully.

In this study, we examined the link between experienced power and psychological aggression within the context of romantic relationships. Participants were asked to indicate the power they have in their romantic relationship and their psychological aggression perpetrated against their partner. Meeting one's core goals and needs may clash with the partner's goals and needs, making conflicts inevitable and positioning power as a relevant predictor of aggression in intimate relationships. Further, we conceptualized psychopathy as a potential moderator, because it is linked to antisocial and callous tendencies. Thus, psychopathy may explain when power relates to psychological aggression. To this end, we conducted two studies. Study 1 included participants in committed romantic relationships. Study 2 included couples to test dyadic effects.

## 1 Power and Aggression

Power is usually understood as the potential to influence others (Anderson et al. 2012; Körner et al. 2025; Simpson et al. 2015) with the goal of attaining core needs and goals (Overall et al. 2023). Importantly, power is a property of a relationship, which is why the relational nature of power is important to consider (Anderson et al. 2012), particularly in intimate relationships (Körner et al. 2025). Moreover, one's *experience* of power is usually a stronger predictor of thoughts, feelings, and behaviors than objective sources of power such as income or social class (Körner and Schütz 2021; Weisfeld et al. 1992). Findings consistently show greater experienced power to be linked to greater behavioral approach, forgiveness, and better relationship quality (Körner and Schütz 2021; Körner et al. 2022; Overall et al. 2023).

Although much research examines the fundamental link between power and different forms of aggression, both theories and empirical studies are inconsistent in their predictions and findings. Theories make predictions on power and aggression in general without specifying the type of relationship or the form of aggression. Approach/Inhibition Theory of Power (Keltner et al. 2003) suggests that power leads to approach‐related behavior such as being less constrained by norms and thus possibly showing antisocial behaviors such as aggression. Similarly, Social Distance Theory of Power (Magee and Smith 2013) proposes that power leads to socially disengaging emotions (e.g., higher anger, lower empathy), which in turn could increase aggression. By contrast, Relative Deprivation Theory (Smith et al. 2012) proposes that individuals experience anger if they feel worse off than others, which could increase aggression. According to this theory, low‐power individuals should behave aggressively because the state of low power is characterized by feeling disadvantaged. Unfortunately, empirical studies on power in romantic relationships do not provide a clear pattern of results either. Whereas several studies reported negative links between power and aggression (Babcock et al. 1993; Cross et al. 2019; Harrington et al. 2021), other studies found a nonsignificant (Toplu‐Demirtaş and Fincham 2022) or positive link (Bentley et al. 2007; Ronfeldt et al. 1998). Further, the link between power and different forms of aggression (e.g., psychological, physical) tended to be consistent within individual studies (e.g., Babcock et al. 1993, and Harrington et al. 2021, found negative links of similar magnitude with both forms), but varied between studies. This mixed pattern of results calls for examining moderating factors.

## 2 Moderating Factors of Power

Power in general is assumed to ignite certain goals and behavioral tendencies that vary depending on personal variables (Guinote 2017). For example, communal (vs. exchange) orientation, person (vs. product) orientation, self (vs. other) focus, and perceived partner responsiveness have been identified as relevant moderators of power effects (Alonso‐Ferres et al. 2021; Chen et al. 2001; Gordon and Chen 2013; Overbeck and Park 2006). More importantly, among working people and by employing experimental power manipulations, researchers have found ego threat to elicit aggression among powerholders, whereas perceived worth has been found to buffer against aggression among powerholders (Fast and Chen 2009; Weick et al. 2018).

Among the moderators primarily studied in the power literature are social orientations and situational factors but to a lesser degree personality variables (but see Macenczak et al. 2016). This is problematic because key power theories propose that personality traits are relevant moderators to understand power effects (Keltner et al. 2003; Guinote 2017). We propose psychopathy as an important moderator. Psychopathy is characterized by a lack of empathy and shallow emotional responses. Individuals scoring high on psychopathy are interpersonally antagonistic, selfish, callous, negligent, and experience high anger (Lynam and Derefinko 2006). Meta‐analyses show a robust positive link between psychopathy and aggression (Hyatt et al. 2019; Muris et al. 2017).

Psychopathy can thus be expected to moderate the link between power and psychological aggression. Yet, two different moderation effects are possible. First, psychopathy may strengthen the link between *high* power and psychological aggression because power ignites personality traits and their behaviors (Guinote 2017). That is, individuals scoring high on psychopathy can be expected to show psychological aggression (Hyatt et al. 2019), and this tendency should be more pronounced when feeling powerful. Second, psychopathy may strengthen the link between *low* power and psychological aggression because individuals high in psychopathy may use psychological aggression in situations where they lack interpersonal control and do not feel in charge. In fact, psychopathy is linked to a higher power motive and the wish to be superior (Čekrlija et al. 2023; Verona et al. 2023). Note that our focus is on high levels of psychopathy because the two opposing theoretical stances argue that high psychopathy makes a difference. We did not expect power to be strongly positively or negatively related to psychological aggression at low levels of psychopathy (though we will report the power‐aggression link at both high and low levels of psychopathy).

## 3 The Present Research

This study had three goals. First, we examined the link between power and psychological aggression to help clarify previously inconclusive findings on how the two variables are linked. We used an established measure of power to find out how powerful participants felt in their relationship (Anderson et al. 2012; Körner et al. 2025). Such an approach avoids the potential problem of confounding actor and partner power—an issue that has been prevalent in the literature on this topic because several studies simply asked participants who has more power (e.g., Babcock et al. 1993). Such an approach makes it impossible to determine whether self‐perceived or partner‐perceived power drives the effects. Moreover, we focused on self‐reported perpetration of psychological aggression because we expected baseline rates of other forms of aggression (physical, sexual) to be low. Thus, studying psychological aggression allows us to focus on relational patterns that may occur in most relationships, including relatively healthy ones that do not involve physical aggression.

Second, we examined both intra‐ and interpersonal links between power and psychological aggression. Both power and aggression are fundamental relational constructs and dyadic power theories call for analyzing mutual influences (Overall et al. 2023; Simpson et al. 2015). Thus, accounting for the relational nature by asking both members of a dyad is necessary to more fully understand how power shapes psychological aggression of both partners.

Third, we examined psychopathy as a potential moderator in the link between power and psychological aggression. Does psychopathy strengthen a link between high power and psychological aggression or a link between low power and psychological aggression? Further, we analyzed both the *intra*personal moderation effect of psychopathy on the link between an actor's power and an actor's psychological aggression, and a variety of *inter*personal moderation effects (e.g., does an actor's psychopathy interact with actor's power in predicting the partner's psychological aggression?).

In both studies, we recruited individuals involved in romantic relationships because psychological aggression has been reported to be more maladaptive in community samples (like couples) than in offender samples (Garofalo et al. 2016, 2018, 2020). In Study 1, individuals currently in romantic relationships participated without their partners. Previous research found that people who participated without their partner reported lower relationship satisfaction and commitment than people who participated as a dyad (Barton et al. 2020). Individuals who participate as a dyad are often those in more satisfied, committed relationships, which may lead to a positively biased sample. Therefore, having participants take part alone is more likely to also capture people with lower relationship satisfaction and thus better reflects the full range of relationship experiences in the general population (DiDonato and Jakubiak 2023). Nevertheless, dyadic data are considered the gold standard in relationship research because they allow for analyzing interpersonal links and accounting for nonindependence (Kenny et al. 2006). Thus, in Study 2, we recruited couples in committed relationships. In both studies, we recruited participants with diverse gender and sexual identities to enhance inclusivity and generalizability. Data, materials, and analysis code are available online at https://osf.io/za2cx/. We report all studies conducted, data exclusions, measures focal to our research question, and all employed analysis methods.

## 4 Study 1

Study 1 examined the direction of the relationship between power and psychological aggression and provided an initial test of the moderating role of psychopathy. We did not formulate specific hypotheses because theory and research suggest that both positive and negative links are possible.

### 4.1 Method

#### 4.1.1 Participants

We collected data from 190 individuals, two were excluded because they completed the survey in an extremely brief amount of time that suggested problems with careful responding (i.e., more than 2 SDs below the average completion time). Participants were 188 individuals 18–80 years old (*M* = 26.52, SD = 10.95) in committed romantic relationships ranging from 1 month to 51 years (*M* = 4.04, SD = 6.49). Of these, 9.6% were married. Most were heterosexual (77.7%), followed by bisexual (16.5%), gay/lesbian (1.9%), and other sexual orientations (3.9%). Participants identified as 61.2% women, 31.4% men, and 7.5% gender diverse. Most participants identified with their gender assigned at birth (88.3%), and some as transgender (11.7%). We assessed achieved power for typical small‐medium effect sizes with this sample size (*α* = 0.05; three tested predictors) using the G*Power software. We had adequate statistical power (0.82) to detect effects of *r* = 0.20.

#### 4.1.2 Procedure

Participants were recruited via university‐intern email lists, social media (i.e., Facebook and WhatsApp groups), and word‐of‐mouth advertising, mainly from Southern Germany (data collection in 2023). The sample comprised a mix of students and working people. Inclusion criteria were: 18 years or older and at least for 1 month in the current romantic relationship. Participants completed an online survey beginning with demographic data and followed by questionnaires on power, psychopathy, and psychological aggression. Survey completion took approximately 10 min. Students received course credit for their participation; others volunteered.

#### 4.1.3 Measures

##### 4.1.3.1 Experienced Power

The 6‐item (e.g., “In my relationship… I think I have a great deal of power”) *Personal Sense of Power Scale* (Anderson et al. 2012; Körner et al. 2022) was employed to assess power within the romantic relationship. This most often used power scale shows good psychometric properties and clearly assesses power in terms of perceived influence over others (Körner et al. 2025). Reliabilities of all measures appear in Table 1.

**Table 1:** Descriptive statistics, reliabilities (McDonald's Omega, Cronbach's Alpha), and correlations (Study 1: Pearson‐correlations; Study 2: Intraclass correlations) across measures.

Variable	Power	Psychopathy	Psychological aggression
Study 1			
Psychopathy	−0.21**	—	—
Psychological aggression	−0.16*	0.13	—
*M*	5.94	2.16	0.24
SD	1.03	0.97	0.21
McDonald's Omega/Cronbach's Alpha	0.78/0.78	0.69/0.69	0.71/0.72
Study 2			
Power	0.13**	−0.25***	−0.32***
Psychopathy	0.01	−0.01	0.20***
Psychological aggression	−0.18***	0.07	0.26***
*M*	6.08	2.25	0.24
SD	0.86	1.01	0.20
McDonald's Omega/Cronbach's Alpha	0.71/0.71	0.78/0.77	0.67/0.67

*Note:* Study 2: The values above the diagonal are correlations within actors. The values below and on the diagonal are correlations across partners (e.g., partner A's and partner B's power). Study 1: *N* = 188 individuals. Study 2: *N* = 226 dyads.

* *p* < 0.05

** *p* < 0.01

*** *p* < 0.001 (two‐tailed).

##### 4.1.3.2 Psychopathy

The 4‐item (e.g., “I tend to be callous or insensitive”) psychopathy subscale of the *Dirty Dozen* (Jonason and Webster 2010) was used to assess psychopathy. This scale was reported to show good discriminant and criterion validity (Jonason and Luévano 2013), although its efficiency comes at the cost of construct coverage (Maples et al. 2014). Nevertheless, we employed this measure because it is one of the most established psychopathy scales, making our findings comparable with those from other studies using this scale. Further, the scale assessed psychopathy in subclinical samples (Jonason and Luévano 2013), like our participants.

##### 4.1.3.3 Psychological Aggression

The 8‐item (e.g., “I threatened to hit or throw something at my partner”) *Revised Conflict Tactics Scale* (Straus et al. 1996) was used to assess psychological aggression.^1^ This is the standard instrument for assessing aggression in intimate relationships (Straus and Douglas 2017). In total, 78.7% of all participants engaged at least in one instance in psychological aggression. Note, again, that we only employed the psychological but not the physical aggression subscale as we expected baseline levels to be much higher for psychological aggression.

### 4.2 Analytic Strategy

First, Pearson correlations were computed. Then, we computed a moderation analysis using model 1 in PROCESS (using mean centering) with power as predictor, psychopathy as moderator, and psychological aggression as outcome. To examine the link between power and psychological aggression at different levels of psychopathy, simple slope analyses were computed for low (−1 SD) and high (+1 SD) levels of the moderator. Analyses controlling for relationship length, relationship status, or age as covariates are presented in the Online Supplement and closely replicated those reported in the manuscript.

### 4.3 Results and Discussion

**Figure 1:**
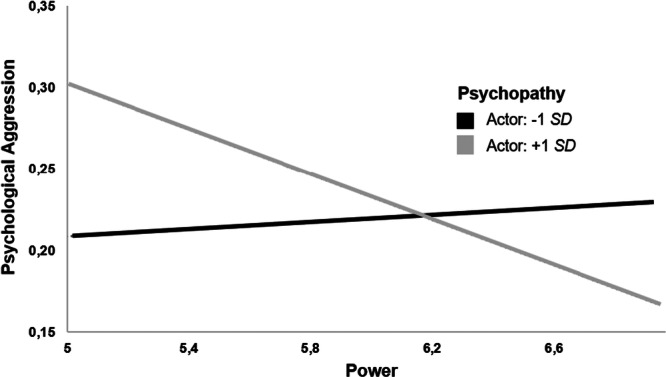
Study 1: The interaction between power and psychopathy on psychological aggression.

Power was negatively related to psychological aggression and psychopathy, whereas psychopathy was positively related to psychological aggression (see Table 1). The moderation analysis showed a significant interaction between power and psychopathy, *b* = −0.04, *p* = 0.012, 95% CI [−0.07, −0.01]. As depicted in Figure 1, simple slope analyses showed no relationship between power and psychological aggression at low levels of psychopathy (−1 *SD, b* = 0.01, *p* = 0.622), and a negative relationship between power and psychological aggression at high levels of psychopathy (+1 SD, *b* = −0.07, *p* = 0.002).

Overall, the present results are in line with studies reporting a negative link between power and aggression (Babcock et al. 1993; Cross et al. 2019). Further, we found that psychopathy moderated the link between power and psychological aggression—in line with our second prediction. Apparently, people scoring high on psychopathy aim at compensating for their lack of power in their relationship with psychological aggression against their partner.

## 5 Study 2

The goal of Study 2 was to replicate and extend the findings from Study 1. In addition, we expected that the pattern found at an intrapersonal level would be found at the interpersonal level, for two possible reasons. First, a high‐power actor may lead their partner to think they are taking charge of the relationship, and they should follow their lead and interaction styles (e.g., low use of psychological aggression). Second, a high‐power actor may make the partner less prone to use conflict‐escalating behaviors such as psychological aggression out of fear of retaliation. Thus, on the interpersonal level, we also predicted a negative link between power and psychological aggression. Further, partner power was reported to promote actors' communal behavior (Overall et al. 2023), which also suggests that partner power may be negatively linked to actors' selfish and antisocial tendencies such as psychological aggression. In an exploratory fashion, we tested how actor's psychopathy moderates the interpersonal power‐aggression link and how partner's psychopathy moderates the intra‐ and interpersonal power‐aggression links.

### 5.1 Method

#### 5.1.1 Participants

Although we collected data from 537 individuals, 85 were excluded because their partners did not participate or they completed the survey in an extremely brief amount of time that suggested problems with careful responding (i.e., more than 2 SDs below the average completion time).^2^ The final dyadic sample thus comprised 226 couples (154 couples in woman‐man relationships and 72 couples in queer relationships) 18 to 90 years old (*M* = 27.29 years, SD = 10.62) who had been in a relationship ranging from 1 month to 37 years (*M* = 3.72 years, SD = 5.25). Of these, 8.8% were married. Most were heterosexual (63.3%), followed by gay/lesbian (18.8%), bisexual (5.5%), pansexual (2.0%), and other sexual orientations (10.4%). Participants identified as 52.7% women, 39.4% men, and 7.3% gender diverse. Most participants identified with their gender assigned at birth (91.6%), and some as transgender (8.4%).

We checked what statistical power would be achieved with this sample when variables were correlated between partners as they were in the present study (α = 0.05; correlation between errors = 0.13; correlations between actor and partner variables = 0.13, Ackerman et al. 2020) and found that we had high statistical power (0.90) to detect actor and partner effects of *β* = 0.15.

#### 5.1.2 Procedure and Measures

As in Study 1, participants were recruited via university‐intern email lists, social media, and word‐of‐mouth advertising, mainly from Southern Germany (data collection in 2024). Inclusion criteria and measures were the same as in Study 1. Participants recruited their partner, who completed the same survey. Each person provided answers independent of their partner. Couple data were linked by using dyad‐specific codes. Participants completed an online survey beginning with demographic data and followed by the same questionnaires on power, psychopathy, and psychological aggression as in Study 1, and some filler items. In total, 78.5% of all participants engaged at least in one instance in psychological aggression. Reliabilities of all scales appear in Table 1. Survey completion took approximately 15 min. The sample comprised a mix of students and working people. Students received course credit for their participation; others volunteered.

#### 5.1.3 Analytic Strategy

First, intraclass correlations (ICCs; see Table 1) were computed using the pairwise correlational method (Kenny et al. 2006). Then, we computed a series of Actor‐Partner Interdependence Models (APIMs) estimated with structural equation modeling (Maximum Likelihood estimation) in M*plus* 8. Consistent with APIM terminology, we use the term “effect,” although it does not imply causality. We modeled both partners' power scores as simultaneous predictors of psychological aggression. The APIM assesses actor (e.g., link between partner A's power and partner A's psychological aggression) and partner effects (e.g., link between partner A's power and partner B's psychological aggression) accounting for the interdependence in measures across partners.

We checked whether gender moderates the link between power and psychological aggression by using the subsample of 154 woman‐man couples. Specifically, we compared the fit of a saturated model (all effects freely estimated) against a model in which actor and partner effects were constrained to be equal across women and men. Because the Likelihood Ratio Test was nonsignificant (*p* > 0.20; Kenny and Ledermann 2010), effects of power on psychological aggression were not moderated by gender (see Online Supplement, https://osf.io/za2cx/?view_only=990c549fbbcb42d4a7247d696287741a). Next, we computed the APIM with all participants (226 couples) and found the actor and partner effects to be very similar to the model with woman‐man couples only. Thus, as neither gender moderated the effects nor the effects changed notably by including queer couples, we analyzed the hypotheses using the full sample.

**Figure 2:**
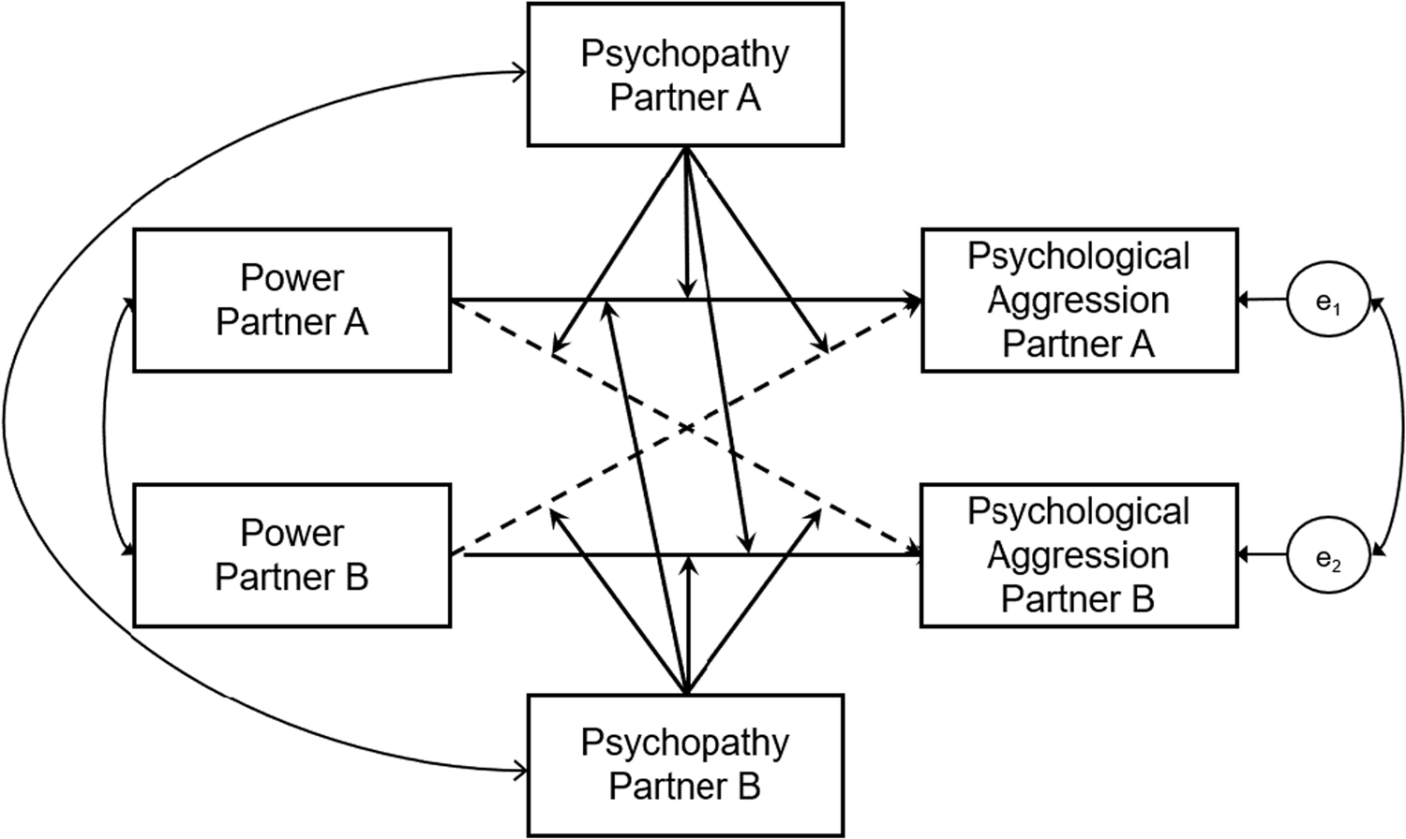
Study 2: Model Specification for the Moderated APIM Estimating the Effect of Power on Psychological Aggression Moderated by Psychopathy. *Note:* Solid paths = actor effects. Dashed paths = partner effects. All predictors of psychological aggression, that is, all power scores, psychopathy scores, and interaction terms (power × psychopathy) were correlated with each other (only partly reflected in the figure).

The main analysis comprised the moderated APIM (Garcia et al. 2015). We computed the full model with power scores of both partners as predictors, psychological aggression scores of both partners as criteria, and psychopathy scores of both partners as moderators for both actor and partner effects (see Figure 2). We grand‐mean centered both the predictor and the moderator before computing the interaction terms (Garcia et al. 2015). To account for the arbitrariness in the assignment of partners within each dyad, we set all corresponding paths, means, intercepts, and (residual) variances equal across partners (Olsen and Kenny 2006). Simple slope analyses were computed for high (+1 SD) and low (−1 SD) levels of psychopathy for each of the four moderation effects. For all coefficients, bootstrapped 95% Confidence Intervals (*k* = 5000 samples) are reported. Analyses controlling for relationship length, relationship status, or age as covariates are presented in the Online Supplement and closely replicated those reported in the manuscript.

### 5.2 Results and Discussion

**Figure 3:**
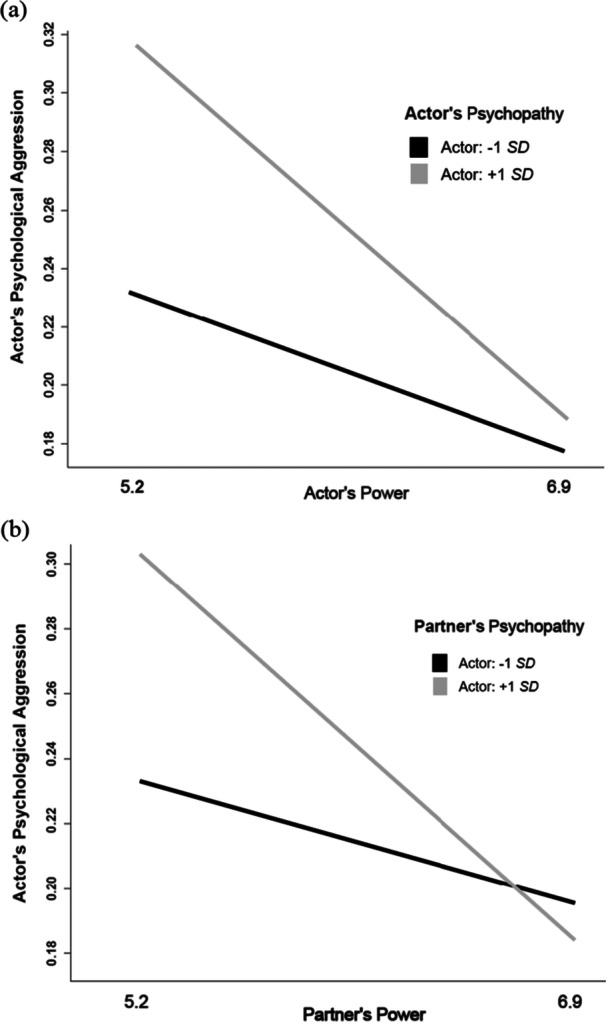
Study 2: The interaction between (a) actor's power and actor's psychopathy and (b) partner's power and partner's psychopathy on actor's psychological aggression.

As in Study 1, actor's power was negatively related to both actor's and partner's psychological aggression, as well as actor's psychopathy, and actor's psychopathy was positively related to actor's psychological aggression (see Table 2). Two out of four moderation effects were significant: First, replicating the results of Study 1, the interaction between actor's power and actor's psychopathy on actor's psychological aggression was significant (actor‐actor interaction, Table 2). As shown in Figure 3, simple slope analyses showed that power was only weakly negatively and non‐significantly linked to psychological aggression at low levels of psychopathy (−1 *SD, b* = −0.03, *p* = 0.066) but was more strongly negatively linked to psychological aggression at high levels of psychopathy (+1 *SD, b* = −0.07, *p* < 0.001). The highest psychological aggression level was reported when actors were low in power but high in psychopathy. At high levels of power, psychological aggression was low for both people scoring relatively low and relatively high on psychopathy.

**Table 2:** Results of the moderated APIM with power as predictor, psychopathy as moderator, and psychological aggression as outcome.

Predictor	Actor effects				Partner effects			
	*β*	95% CI	*SE*	*p*	*β*	95% CI	*SE*	*p*
Power	**−0.23**	[−0.32, −0.13]	0.05	< 0.001	**−0.20**	[−0.28, −0.11]	0.04	< 0.001
Psychopathy	**0.13**	[0.04, 0.21]	0.04	0.004	0.08	[−0.02, 0.15]	0.04	0.076
Interactions	*β*	95% CI	*SE*	*p*				
Actor‐actor	**−0.11**	[−0.20, −0.02]	0.05	0.022	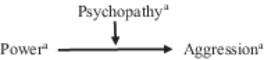			
−1 SD	−0.03	[−0.07, 0.00]	0.02	0.066				
+1 SD	**−0.07**	[−0.11, −0.05]	0.02	< 0.001				
Actor‐partner	−0.10	[−0.20, 0.02]	0.05	0.070	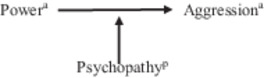			
−1 SD	−0.03	[−0.06, 0.00]	0.02	0.067				
+1 SD	**−0.08**	[−0.12, −0.04]	0.02	< 0.001				
Partner‐partner	**−0.12**	[−0.20, −0.03]	0.04	0.006	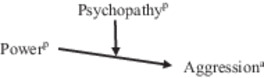			
−1 SD	−0.02	[−0.05, 0.01]	0.01	0.120				
+1 SD	**−0.07**	[−0.09, −0.05]	0.01	< 0.001				
Partner‐actor	−0.05	[−0.14, 0.04]	0.04	0.254	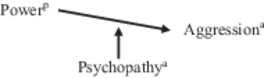			
−1 SD	**−0.03**	[−0.06, −0.01]	0.01	0.017				
+1 SD	**−0.06**	[−0.09, −0.03]	0.02	< 0.001				

*Note:* ^a^ = actor variable. ^p^ = partner variable. Simple slope coefficients (values at −1 SD, +1 SD) are not standardized. The bold values indicate significant *b* coefficients. The significance (exact *p*‐values) of the *b* values can be found in the columns entitled *p*.

Second, the interaction between partner's power and partner's psychopathy on actor's psychological aggression was significant (partner‐partner interaction, Table 2). As shown in Figure 3, simple slope analyses showed that power was not significantly linked to psychological aggression at low levels of psychopathy (−1 *SD, b* = −0.02, *p* = 0.120) but was significantly negatively linked to psychological aggression at high levels of psychopathy (+1 *SD, b* = −0.07, *p* < 0.001). The highest psychological aggression level by actors was reported when partners were low in power but high in psychopathy. At high levels of partner's power, actor's psychological aggression was low for both partners scoring relatively low and relatively high on psychopathy.

Overall, the results from Study 2 replicate and extend the results from Study 1. First, and also consistent with other research (Babcock et al. 1993; Cross et al. 2019), we found that actor power was negatively related to actor's perpetration of psychological aggression. Extending this link to the interpersonal level, we also found actor power to be negatively related to partner's psychological aggression. Second, we found that actor's psychopathy moderated the effect of actor's power on actor's psychological aggression such that psychopathy strengthened the link between low power and psychological aggression. In addition, we found that partner's psychopathy moderated the effect of partner's power on actor's psychological aggression. This finding illustrates a dyadic moderation effect, so that psychological aggression is not only shaped by actor's own power and psychopathy but also by their partners' power and psychopathy.

## 6 General Discussion

To the best of our knowledge, this study provides the first analysis of how psychopathy affects the link between two basic relational constructs: power and psychological aggression. Power and aggression have often been postulated to be intertwined (Weick 2020), and indeed, we found strong links between these variables. In line with Relative Deprivation Theory (Smith et al. 2012), power was negatively linked to psychological aggression across the two studies. Experiencing low power in one's romantic relationship, which is a significant relationship for most people, may lead to a feeling of disadvantage, helplessness, and vulnerability, which in turn can make individuals more likely to resort to psychological aggression. This finding adds to the literature that reports a negative power‐aggression link in intimate relationships (e.g., Cross et al. 2019). Previous research that found a positive power‐aggression link often used problematic measures that conflate actor and partner power by asking participants who has more power in the relationship (“I” vs. “my partner;” Bentley et al. 2007; Ronfeldt et al. 1998). For example, a couple in which both partners experience high power would be considered equivalent to a couple in which both partners experience low power. Also, a person who experiences low power but has a partner who experiences very low power would be considered ‘high power’. Such an approach leaves it unclear whether high actor or low partner power (respectively vice versa) drives the effect (Körner and Schütz 2025; Overall et al. 2023). Thus, future studies in this domain may benefit from employing power measures that differentiate between actor and partner power by asking participants to indicate their absolute power rather than their relative power.

Next, we found greater actor power to be associated with lower partner's psychological aggression. This dyadic effect adds to the literature on how actor power predicts partner outcomes in romantic relationships (Körner and Schütz 2025; Overall et al. 2023). There may be two possible explanations for the present partner effect: First, partners of powerful actors may behave submissively (Tiedens and Fragale 2003) and feel restricted in their ability to act (Keltner et al. 2003), consequently, they do not resort to psychological aggression. However, this seems unlikely as power is often positively correlated within couples (Körner and Schütz 2021; see also Study 2's within‐couple power correlation in Table 1). Second, and more likely, powerful actors may prompt communal behaviors in partners (Overall et al. 2023), perhaps because power in intimate contexts is more likely to be construed and perceived as responsibility. Thus, actors do not engage in dysfunctional behaviors such as psychological aggression if the partner reports high power because partner power may be perceived as taking responsibility for the relationship.

Finally, we found psychopathy to moderate the link between power and psychological aggression (though our sample size may be considered small for detecting moderation effects). In both studies, low power coupled with high psychopathy was the toxic combination that increased psychological aggression. Additionally, in Study 2, we found partner's low power coupled with partner's high psychopathy was also a toxic combination for actor's psychological aggression. Psychopathy is associated with an elevated desire for power and superiority (Čekrlija et al. 2023; Verona et al. 2023). People with an antisocial and callous personality (i.e., high in psychopathy) who feel powerless in a close relationship (Clark and Mills 2011) may compensate for their lack of power via aggression. Interestingly, the aggressive tendencies of individuals with low power and high psychopathy extended to their romantic partners' behaviors, likely due to the strong interdependence within such relationships (Sels et al. 2020). Thus, a dysfunctional relationship dynamic may emerge where both partners engage in psychological aggression (see the significant *r* = 0.26 for psychological aggression in Table 1). In addition, the present findings add to the literature on the link between power and psychopathy. Our results suggest that people who feel powerful in their romantic relationship tend not to have an antisocial or callous personality, supporting the notion that sense of power reflects a socially valued attribute in relationships (Körner and Schütz 2021; Overall et al. 2023). The findings are in line with a study reporting negative or mixed links between sense of power and various psychopathy measures (Verona et al. 2023).

### 6.1 Theoretical Advances and Implications

This study provides evidence that psychopathy as a personality trait is an important moderator in the link between power and psychological aggression. Past research has typically tested social orientations and situational factors as variables moderating the link between power and outcome variables (e.g., Chen et al. 2001; Overbeck and Park 2006; Pietromonaco et al. 2021). Personality traits such as psychopathy were barely considered in past research, although key power theories suggest that personality traits moderate how power affects outcomes (e.g., Keltner et al. 2003; Guinote 2017). We expect that psychopathy may also be relevant to understand when power is linked to other antisocial behaviors (e.g., conflict, infidelity, dishonesty, exploitation). More broadly, power theories may provide more detailed predictions when considering personality traits, such as psychopathy, as moderating variables.

Our findings also indicate that the moderation effect of psychopathy affects psychological aggression of low power (but not high power) actors. More specifically, low‐power people differed in their level of self‐reported psychological aggression depending on their psychopathy, whereas high‐power people did not. This differs from past research, which has focused on how *high*‐power people differ in their thoughts and behaviors depending on moderators (e.g., Fast and Chen 2009; Overbeck and Park 2006). Also, power is assumed to energize thoughts, feelings, and behavior depending on goals and predispositions, which suggests that people differ more from each other the higher their power is (Guinote 2017). However, here we were able to show for the first time that the moderation effect of psychopathy is relevant with low power. This finding aligns with initial evidence suggesting that moderators affect the link between low power and specific outcomes (Harrington et al. 2021; Overall et al. 2016). Thus, future power theories and empirical studies should be careful not to overlook how moderators affect low power (see also Schaerer et al. 2018).

These findings are the first to show a moderating variable affects the link between power and a partner variable (i.e., psychological aggression). Indeed, power is a socio‐relational construct that has been both theoretically postulated and empirically found to be associated with partner variables (Körner and Schütz 2021; Overall et al. 2023; Simpson et al. 2015). However, past research has not tested whether power interacts with certain variables (e.g., personality traits) in predicting partner outcomes. Our research can thus enrich both dyadic power theories as well as power theories proposing moderators by including dyadic moderators. We assume that (a) psychopathy may moderate the link between actor's power and other dysfunctional partner variables and (b) that other relational predispositions (e.g., humility, attachment) may be additional relevant candidates for moderating the link between actor's power and partner's aggression. Thus, future studies on power may benefit from dyadic designs to study not only intra‐ and interpersonal associations but also dyadic moderation effects.

Finally, we found that the links between power and psychological aggression were similar across genders (women, men) and sexual orientations (straight, queer). This may suggest that power works in similar ways in different close relationship types. Past research on power mainly studied people in heterosexual relationships, ignoring other types of relationships. The limited literature on power in same‐sex couples, for example, has mostly focused on whether these relationships are characterized by perceptions of equal power but not how power is linked to other variables (Peplau and Fingerhut 2007). Our research adds to the limited knowledge base surrounding power dynamics among queer individuals. Future studies on both social power and aggression may benefit from including queer participants.

### 6.2 Limitations and Future Research

Despite identifying both intra‐ and interpersonal links, including moderation, our cross‐sectional data limit causal conclusions. Power theories propose power as an antecedent of behaviors such as aggression (Keltner et al. 2003; Simpson et al. 2015) and empirical studies also conceive power as a predictor of aggression (Fast and Chen 2009; Weick et al. 2018). In fact, studies have shown power‐related variables to be a stronger precursor of aggression than vice versa (Cillessen and Mayeux 2004). Future longitudinal and experimental studies could tackle the question of causality—ideally with very large sample sizes that allow the detection of even small moderation effects.

Further, we only studied self‐reported perpetration of psychological aggression. In fact, psychological aggression was reported to be much more prevalent in intimate relationships than physical aggression (Graña Gómez and Cuenca Montesino 2014). Nevertheless, physical and sexual forms of aggression (Straus et al. 1996) and victimization should also be studied along with the functions that various types of aggression serve (Hayes and Anderson 2024) to expand the scope of antisocial behaviors and perspectives in couples. In addition, other contexts (e.g., work, school) could also be studied to increase generalizability.

Another limitation pertains to the psychopathy measure we used. The Dirty Dozen is a well‐established instrument and thus helpful for building an accumulative instead of a fragmented psychological science (Anvari et al. 2025), however, the psychopathy subscale has been criticized for not fully capturing the construct (Miller et al. 2012). Therefore, replication studies are needed that employ longer psychopathy scales with broader construct coverage (e.g., the Levenson Self‐Report Psychopathy Scale, Levenson et al. 1995, or the Psychopathic Personality Inventory‐Revised, Lilienfeld 2005).

Finally, despite studying not only heterosexual couples but also LGBTQ+ individuals in romantic relationships, the current study is restricted in its generalizability to participants from a Western and industrialized country (Henrich et al. 2010). Power is construed and valued differently across cultures (Torelli et al. 2020). As such, future studies could test the generalizability of the findings by studying couples from collectivistic and honor cultures.

## 7 Conclusion

Power and aggression are core variables pervading every social relationship. We found greater power to be associated with both lower actor and lower partner psychological aggression in individuals in romantic relationships. Delineating a more nuanced picture of this relationship, we identified psychopathy as a relevant variable affecting the power‐aggression link. The most toxic combination for relationships, that is, highest self‐reported psychological aggression by *both* partners, was found when one individual reported low power and high psychopathy. These findings add to the literature concerning the power‐aggression link and advance existing power theories by highlighting how personality traits such as psychopathy affect both intra‐ and interpersonal links between power and outcome variables.

## Author Contributions

Conception and design of the work: Robert Körner. Data collection and analysis: Robert Körner. Drafting the manuscript: Robert Körner. Critical revision of the manuscript: Robert Körner, Astrid Schütz, Brad J. Bushman. Resources: Astrid Schütz.

## Ethics Statement

All procedures performed in studies involving human participants were in accordance with the ethical standards of the institutional and/or national research committee and with the 1964 Helsinki declaration and its later amendments or comparable ethical standards. This article does not contain any studies with animals performed by any of the authors.

## Conflicts of Interest

The authors declare no conflicts of interest.

## Supporting information


**Table S1:** Results of APIM Analyses Estimating the Actor and Partner Associations between Experienced Power and Psychological aggression.
**Table S2:** Results of the Moderated APIM With Power as Predictor, Psychopathy as Moderator, and Verbal Aggression as Outcome.
**Table S3:** Results of the Moderated APIM: Power (Predictor), Psychopathy (Moderator), Aggression (Outcome), Relationship Duration (Control Variables).
**Table S4:** Results of the Moderated APIM: Power (Predictor), Psychopathy (Moderator), Aggression (Outcome), Relationship Status (Control Variables).
**Table S5:** Results of the Moderated APIM: Power (Predictor), Psychopathy (Moderator), Aggression (Outcome), Age (Control Variables).

## Data Availability

The data that support the findings of this study are openly available in OSF at https://osf.io/za2cx/.

## Endnotes

1 Because the data were highly skewed, we used a poly‐perpetration scoring (S. Hamby, personal communication, November 4, 2024; Straus and Douglas 2017). That is, all eight items were recoded to whether a specific aggressive act happened or did not, and then the total score was obtained by averaging these responses.

2 Excluded participants did not differ from participants of the final sample in any of our key variables (power, psychological aggression, psychopathy; see Online Supplement).
