# Supplementary material for: Low Power and High Psychopathy: A Toxic Combination for Psychological Aggression
Source: Aggress Behav. 2025 Aug 6;51(5):e70045. doi: 10.1002/ab.70045 (PMC12326235; doi:10.1002/ab.70045)
Supplement: Supplementary file 1 — Table S1: Results of APIM Analyses Estimating the Actor and Partner Associations between Experienced Power and Psychological aggression. Table S2: Results of the Moderated APIM With Power as Predictor, Psychopathy as Moderator, and Verbal Aggression as Outcome. Table S3: Results of the Moderated APIM: Power (Predictor), Psychopathy (Moderator), Aggression (Outcome), Relationship Duration (Control Variables). Table S4: Results of the Moderated APIM: Power (Predictor), Psychopathy (Moderator), Aggression (Outcome), Relationship Status (Control Variables). Table S5: Results of the Moderated APIM: Power (Predictor), Psychopathy (Moderator), Aggression (Outcome), Age (Control Variables). [file AB-51-e70045-s001.docx]

**ONLINE SUPPLEMENT**

**to**

**Low Power and High Psychopathy:**

**A Toxic Combination for Psychological Aggression**

Overview

[Scales and Items Used to Assess Power, Psychopathy, and Aggression (Studies 1, 2) 2](#_Toc203576065)

[Results of Likelihood Ratio Tests Examining Gender as a Moderator (Study 2) 3](#_Toc203576066)

[Comparison Excluded Cases with Final Sample (Study 2) 4](#_Toc203576067)

[Additional Analyses: Subsamples (Study 2) 5](#_Toc203576068)

[Additional Analyses: Verbal Aggression Items (Studies 1-2) 6](#_Toc203576069)

[Additional Analyses: Sociodemographic Controls (Studies 1-2) 8](#_Toc203576070)

# Scales and Items Used to Assess Power, Psychopathy, and Aggression (Studies 1, 2)

Experienced Power:

Instruction: “In the relationship with my partner….”

1. I can get my partner to listen to what I say.
2. My wishes do not carry much weight.
3. Even if I voice them, my views have little sway.
4. I think I have a great deal of power.
5. My ideas and opinions are often ignored.
6. Even when I try, I am not able to get my way.

1 = disagree strongly, 2 = disagree, 3 = disagree a little, 4 = neither agree nor disagree, 5 = agree a little, 6 = agree, 7 = agree strongly

(Note that the validated German version of the Personal Sense of Power Scale contains 6 items, compared to 8 items in the English version.)

Psychopathy:

1. I tend to lack remorse.
2. I tend to be unconcerned with the morality of my actions.
3. I tend to be callous or insensitive.
4. I tend to be cynical.

1 = disagree strongly, 2 = disagree, 3 = disagree a little, 4 = neither agree nor disagree, 5 = agree a little, 6 = agree, 7 = agree strongly

Psychological Aggression

1. I insulted or swore at my partner.
2. I shouted or screamed at my partner.
3. I stormed out of the room, the house or the yard during a disagreement.
4. I said something to annoy my partner.
5. I called my partner fat or ugly.
6. I destroyed something that belongs to my partner.
7. I accused my partner of being a bad lover.
8. I threatened to hit or throw something at my partner.

1 = never, 2 = once, 3 = twice, 4 = 3-5 times, 5 = 6-10 times, 6 = 11-20 times, 7 = > 20 times, 8 = not in referent period but happened before

# Results of Likelihood Ratio Tests Examining Gender as a Moderator (Study 2)

To test significance of the Likelihood Ratio Test, a conservative *p*-value of .20 was chosen (Kenny & Ledermann, 2010). The Likelihood Ratio Test examining the moderating role of gender among straight couples (*N* = 154) was not significant, χ²(2) = 2.111, *p* = .348. Thus, the equal-actor-equal-partner effects model was favored. In other words, the associations between power and psychological aggression are not moderated by gender for the straight couples. Based on this finding, setting actor and partner effects equal across women and men and thus treating dyads as indistinguishable (as with the queer participants) is justified.

# Comparison Excluded Cases with Final Sample (Study 2)

Participants who were excluded because their partners did not complete the survey or because they rushed through the survey (*N* = 85) did not significantly differ from participants who were in the final sample (*N* = 452) with respect to power (*M*_final sample_ = 6.08, *SD* = 0.86, *M*_excluded cases_ = 5.86, *SD* = 1.19), *t*(534) = 1.99, *p* = .112, psychological aggression (*M*_final sample_ = 0.24, *SD* = 0.20, *M*_excluded cases_ = 0.28, *SD* = 0.22), *t*(534) = -1.45, *p* = .147, or psychopathy (*M*_final sample_ = 2.25, *SD* = 1.01, *M*_excluded cases_ = 2.02, *SD* = 0.94), *t*(533) = 1.90, *p* = .058, *d* = 0.22.

# Additional Analyses: Subsamples (Study 2)

**Table S1**

*Results of APIM Analyses Estimating the Actor and Partner Associations between Experienced Power and Psychological aggression*

|  |  | Actor Power | | | | Partner Power | | | | | |  |
| --- | --- | --- | --- | --- | --- | --- | --- | --- | --- | --- | --- | --- |
| Outcome |  | β | 95% CI | *SE* | *p* | |  | β | 95% CI | *SE* | *p* | |
| Model 1: Only Straight Couples (*N* = 154) | | | |  |  | |  |  |  |  |  | |
| Psychological Aggression |  | **-0.24** | [-0.35, -0.13] | 0.06 | < .001 | |  | **-0.25** | [-0.38, -0.12] | 0.07 | < .001 | |
| Model 2: Only Queer Couples (*N* = 72) | | | |  |  | |  |  |  |  |  | |
| Psychological Aggression |  | **-0.27** | [-0.41, -0.14] | 0.07 | < .001 | |  | **-0.22** | [-0.36, -0.07] | 0.07 | .002 | |
| Model 3: All Couples (*N* = 226) | | | | | | | | | | | | |
| Psychological Aggression |  | **-0.23** | [-0.31, -0.16] | 0.04 | < .001 | |  | **-0.25** | [-0.35, -0.15] | 0.05 | < .001 | |

*Note.* *b* = Unstandardized regression coefficient, CI = Bootstrapped 95% confidence interval. The bold values indicate significant *b* coefficients (*p* < .01).

The results highlight that the standardized regression coefficients did barely differ between the straight (Model 1) and the queer couples (Model 2). Also, merging the subsamples leads to very similar standardized regression coefficients (see Model 3). Results are significant in all three models.

# Additional Analyses: Verbal Aggression Items (Studies 1-2)

A reviewer asked us to repeat the analyses using the verbal aggression items from the CTS-2. To address this, we created a composite variable labeled “verbal aggression” based on items 1, 2, 4, 5, and 7, which clearly reflect behaviors involving insults and expressions of anger. While this variable has a narrower scope than the broader psychological aggression construct, it specifically captures verbal aggression.

Study 1: The moderation analysis showed a significant interaction between power and psychopathy, *b* = -0.04, *p* = .039, 95% CI [-0.07, -0.002]. Simple slope analyses showed no relationship between power and psychological aggression at low levels of psychopathy (-1 *SD, b* = 0.01, *p* = .662), and a negative relationship between power and psychological aggression at high levels of psychopathy (+1 *SD*, *b* = -0.05, *p* = .014). Thus, the main result was replicated when using only the verbal aggression items.

Study 2: Again, the results (see Table S2) closely replicated those reported in the manuscript.

**Table S2**

*Results of the Moderated APIM With Power as Predictor, Psychopathy as Moderator, and Verbal Aggression as Outcome*

| Predictor |  | Actor effects | | | |  | Partner effects | | | |
| --- | --- | --- | --- | --- | --- | --- | --- | --- | --- | --- |
|  |  | β | 95% CI | *SE* | *p* |  | β | 95% CI | *SE* | *p* |
| Power |  | **-0.20** | [-0.29, -0.10] | 0.05 | < .001 |  | **-0.17** | [-0.25, -0.08] | 0.04 | < .001 |
| Psychopathy |  | **0.15** | [0.06, 0.24] | 0.04 | .001 |  | **0.10** | [0.01, 0.18] | 0.04 | .016 |
|  |  |  |  |  |  |  |  |  |  |  |
| Interactions |  | β | 95% CI | *SE* | *p* |  |  |  |  |  |
| Actor-actor |  | **-0.11** | [-0.20, -0.03] | 0.05 | .011 |  |  | Psychopathy^a^ |  |  |
|  |  |  |  |  |  |  |  |  |  |  |
| -1 *SD* |  | -0.03 | [-0.07, 0.01] | 0.02 | .145 |  | Power^a^ |  | Aggression^a^ |  |
| +1 *SD* |  | **-0.08** | [-0.12, -0.05] | 0.02 | < .001 |  |  |  |  |  |
|  |  |  |  |  |  |  |  |  |  |  |
|  |  |  |  |  |  |  |  |  |  |  |
| Actor-partner |  | -0.10 | [-0.19, 0.01] | 0.05 | .054 |  | Power^a^ |  | Aggression^a^ |  |
|  |  |  |  |  |  |  |  |  |  |  |
| -1 *SD* |  | -0.03 | [-0.06, 0.01] | 0.02 | .128 |  |  |  |  |  |
| +1 *SD* |  | **-0.09** | [-0.14, -0.04] | 0.03 | < .001 |  |  | Psychopathy^p^ |  |  |
|  |  |  |  |  |  |  |  |  |  |  |
|  |  |  |  |  |  |  |  |  |  |  |
| Partner-partner |  | **-0.13** | [-0.22, -0.04] | 0.05 | .005 |  |  | Psychopathy^p^ |  |  |
|  |  |  |  |  |  |  |  |  |  |  |
| -1 *SD* |  | -0.02 | [-0.05, 0.02] | 0.02 | .382 |  | Power^p^ |  |  |  |
| +1 *SD* |  | **-0.08** | [-0.11, -0.05] | 0.02 | < .001 |  |  |  | Aggression^a^ |  |
|  |  |  |  |  |  |  |  |  |  |  |
|  |  |  |  |  |  |  |  |  |  |  |
| Partner-actor |  | -0.06 | [-0.15, 0.02] | 0.04 | .138 |  | Power^p^ |  |  |  |
|  |  |  |  |  |  |  |  |  | Aggression^a^ |  |
| -1 *SD* |  | -0.03 | [-0.06, 0.00] | 0.02 | .100 |  |  |  |  |  |
| +1 *SD* |  | **-0.07** | [-0.11, -0.03] | 0.02 | .001 |  |  | Psychopathy^a^ |  |  |

*Notes.* ^a^ = actor variable. ^p^ = partner variable. Simple slope coefficients (values at -1 *SD*, +1 *SD*) are not standardized.

# Additional Analyses: Sociodemographic Controls (Studies 1-2)

Guided by comments during the review process, we conducted analyses examining whether the links between power and psychological aggression moderated by psychopathy were robust to relationship length, relationship status, and participants’ age.

**Results for Relationship Length** (measured in months)

Study 1: The moderation analysis showed a significant interaction between power and psychopathy, *b* = -0.04, *p* = .015, 95% CI [-0.07, -0.01] – also after controlling for relationship length. Simple slope analyses showed no relationship between power and psychological aggression at low levels of psychopathy (-1 *SD, b* = 0.01, *p* = .704), and a negative relationship between power and psychological aggression at high levels of psychopathy (+1 *SD*, *b* = -0.06, *p* = .002).

Study 2: We repeated the moderated APIM analyses detailed in the manuscript (Study 2, Analytic Strategy) and added relationship length as a predictor of psychological aggression for both partners. The results are displayed in Table S3 and closely replicated those reported in the manuscript.

**Results for Relationship Status** (2-level category: “serious relationship” vs. “married”)

Study 1: After controlling for relationship status the moderation analysis also showed a significant interaction between power and psychopathy, *b* = -0.04, *p* = .030, 95% CI [-0.07, -0.004]. Simple slope analyses showed no relationship between power and psychological aggression at low levels of psychopathy (-1 *SD, b* = 0.01, *p* = .584), and a negative relationship between power and psychological aggression at high levels of psychopathy (+1 *SD*, *b* = -0.06, *p* = .016).

Study 2: We repeated the moderated APIM analyses detailed in the manuscript (Study 2, Analytic Strategy) and added relationship status as a predictor of psychological aggression for both partners. The results are displayed in Table S4 and closely replicated those reported in the manuscript.

**Age** (measured in years)

Study 1: After controlling for age, the moderation analysis also showed a significant interaction between power and psychopathy, *b* = -0.04, *p* = .011, 95% CI [-0.08, -0.01]. Simple slope analyses showed no relationship between power and psychological aggression at low levels of psychopathy (-1 *SD, b* = 0.01, *p* = .653), and a negative relationship between power and psychological aggression at high levels of psychopathy (+1 *SD*, *b* = -0.07, *p* = .002).

Study 2: We repeated the moderated APIM analyses detailed in the manuscript (Study 2, Analytic Strategy) and added age as a predictor of psychological aggression for both partners. The results are displayed in Table S5 and closely replicated those reported in the manuscript.

**Table S3**

*Results of the Moderated APIM: Power (Predictor), Psychopathy (Moderator), Aggression (Outcome), Relationship Duration (Control Variables)*

| Predictor |  | Actor effects | | | |  | Partner effects | | | |
| --- | --- | --- | --- | --- | --- | --- | --- | --- | --- | --- |
|  |  | β | 95% CI | *SE* | *p* |  | β | 95% CI | *SE* | *p* |
| Power |  | **-0.21** | [-0.30, -0.11] | 0.05 | < .001 |  | **-0.17** | [-0.26, -0.08] | 0.04 | < .001 |
| Psychopathy |  | **0.14** | [0.05, 0.22] | 0.04 | .002 |  | **0.09** | [-0.01, 0.16] | 0.04 | .035 |
| Relationship duration |  | 0.02 | [-0.61, 3.58] | 0.02 | .982 |  | 0.09 | [-0.38, 3.34] | 0.08 | .934 |
|  |  |  |  |  |  |  |  |  |  |  |
| Interactions |  | β | 95% CI | *SE* | *p* |  |  |  |  |  |
| Actor-actor |  | **-0.11** | [-0.20, -0.02] | 0.05 | .016 |  |  | Psychopathy^a^ |  |  |
|  |  |  |  |  |  |  |  |  |  |  |
| -1 *SD* |  | -0.03 | [-0.06, 0.01] | 0.02 | .129 |  | Power^a^ |  | Aggression^a^ |  |
| +1 *SD* |  | **-0.07** | [-0.10, -0.04] | 0.02 | < .001 |  |  |  |  |  |
|  |  |  |  |  |  |  |  |  |  |  |
|  |  |  |  |  |  |  |  |  |  |  |
| Actor-partner |  | -0.10 | [-0.19, 0.02] | 0.05 | .057 |  | Power^a^ |  | Aggression^a^ |  |
|  |  |  |  |  |  |  |  |  |  |  |
| -1 *SD* |  | -0.02 | [-0.06, 0.00] | 0.02 | .137 |  |  |  |  |  |
| +1 *SD* |  | **-0.07** | [-0.11, -0.03] | 0.02 | .001 |  |  | Psychopathy^p^ |  |  |
|  |  |  |  |  |  |  |  |  |  |  |
|  |  |  |  |  |  |  |  |  |  |  |
| Partner-partner |  | **-0.12** | [-0.21, -0.03] | 0.04 | .005 |  |  | Psychopathy^p^ |  |  |
|  |  |  |  |  |  |  |  |  |  |  |
| -1 *SD* |  | -0.01 | [-0.04, 0.01] | 0.01 | .307 |  | Power^p^ |  |  |  |
| +1 *SD* |  | **-0.06** | [-0.09, -0.04] | 0.01 | < .001 |  |  |  | Aggression^a^ |  |
|  |  |  |  |  |  |  |  |  |  |  |
|  |  |  |  |  |  |  |  |  |  |  |
| Partner-actor |  | -0.05 | [-0.14, 0.04] | 0.04 | .266 |  | Power^p^ |  |  |  |
|  |  |  |  |  |  |  |  |  | Aggression^a^ |  |
| -1 *SD* |  | -0.03 | [-0.05, -0.00] | 0.01 | .052 |  |  |  |  |  |
| +1 *SD* |  | **-0.05** | [-0.08, -0.02] | 0.02 | .002 |  |  | Psychopathy^a^ |  |  |

*Notes.* ^a^ = actor variable. ^p^ = partner variable. Simple slope coefficients (values at -1 *SD*, +1 *SD*) are not standardized.

**Table S4**

*Results of the Moderated APIM: Power (Predictor), Psychopathy (Moderator), Aggression (Outcome), Relationship Status (Control Variables)*

| Predictor |  | Actor effects | | | |  | Partner effects | | | |
| --- | --- | --- | --- | --- | --- | --- | --- | --- | --- | --- |
|  |  | β | 95% CI | *SE* | *p* |  | β | 95% CI | *SE* | *p* |
| Power |  | **-0.22** | [-0.32, -0.12] | 0.05 | < .001 |  | **-0.19** | [-0.27, -0.11] | 0.04 | < .001 |
| Psychopathy |  | **0.13** | [0.04, 0.21] | 0.04 | .003 |  | 0.08 | [-0.01, 0.15] | 0.04 | .071 |
| Relationship status |  | 0.00 | [-0.11, 0.10] | 0.05 | .935 |  | -0.01 | [-0.15, 0.02] | 0.04 | .134 |
|  |  |  |  |  |  |  |  |  |  |  |
| Interactions |  | β | 95% CI | *SE* | *p* |  |  |  |  |  |
| Actor-actor |  | **-0.11** | [-0.20, -0.02] | 0.05 | .022 |  |  | Psychopathy^a^ |  |  |
|  |  |  |  |  |  |  |  |  |  |  |
| -1 *SD* |  | -0.03 | [-0.06, 0.00] | 0.02 | .129 |  | Power^a^ |  | Aggression^a^ |  |
| +1 *SD* |  | **-0.07** | [-0.10, -0.05] | 0.02 | < .001 |  |  |  |  |  |
|  |  |  |  |  |  |  |  |  |  |  |
|  |  |  |  |  |  |  |  |  |  |  |
| Actor-partner |  | -0.10 | [-0.20, 0.01] | 0.05 | .057 |  | Power^a^ |  | Aggression^a^ |  |
|  |  |  |  |  |  |  |  |  |  |  |
| -1 *SD* |  | -0.02 | [-0.06, 0.00] | 0.02 | .137 |  |  |  |  |  |
| +1 *SD* |  | **-0.07** | [-0.12, -0.04] | 0.02 | .001 |  |  | Psychopathy^p^ |  |  |
|  |  |  |  |  |  |  |  |  |  |  |
|  |  |  |  |  |  |  |  |  |  |  |
| Partner-partner |  | **-0.12** | [-0.20, -0.03] | 0.04 | .007 |  |  | Psychopathy^p^ |  |  |
|  |  |  |  |  |  |  |  |  |  |  |
| -1 *SD* |  | -0.01 | [-0.05, 0.01] | 0.01 | .307 |  | Power^p^ |  |  |  |
| +1 *SD* |  | **-0.06** | [-0.09, -0.04] | 0.01 | < .001 |  |  |  | Aggression^a^ |  |
|  |  |  |  |  |  |  |  |  |  |  |
|  |  |  |  |  |  |  |  |  |  |  |
| Partner-actor |  | -0.05 | [-0.14, 0.03] | 0.04 | .229 |  | Power^p^ |  |  |  |
|  |  |  |  |  |  |  |  |  | Aggression^a^ |  |
| -1 *SD* |  | -0.03 | [-0.06, -0.01] | 0.01 | .052 |  |  |  |  |  |
| +1 *SD* |  | **-0.05** | [-0.09, -0.03] | 0.02 | .002 |  |  | Psychopathy^a^ |  |  |

*Notes.* ^a^ = actor variable. ^p^ = partner variable. Simple slope coefficients (values at -1 *SD*, +1 *SD*) are not standardized.

**Table S5**

*Results of the Moderated APIM: Power (Predictor), Psychopathy (Moderator), Aggression (Outcome), Age (Control Variables)*

| Predictor |  | Actor effects | | | |  | Partner effects | | | |
| --- | --- | --- | --- | --- | --- | --- | --- | --- | --- | --- |
|  |  | β | 95% CI | *SE* | *p* |  | β | 95% CI | *SE* | *p* |
| Power |  | **-0.22** | [-0.31, -0.12] | 0.05 | < .001 |  | **-0.19** | [-0.27, -0.10] | 0.04 | < .001 |
| Psychopathy |  | **0.13** | [0.05, 0.22] | 0.04 | .002 |  | 0.08 | [-0.01, 0.15] | 0.04 | .059 |
| Age |  | -0.02 | [-0.20, 0.15] | 0.09 | .825 |  | 0.08 | [-0.10, 0.25] | 0.09 | .353 |
|  |  |  |  |  |  |  |  |  |  |  |
| Interactions |  | β | 95% CI | *SE* | *p* |  |  |  |  |  |
| Actor-actor |  | **-0.11** | [-0.20, -0.02] | 0.05 | .017 |  |  | Psychopathy^a^ |  |  |
|  |  |  |  |  |  |  |  |  |  |  |
| -1 *SD* |  | -0.03 | [-0.06, 0.01] | 0.02 | .097 |  | Power^a^ |  | Aggression^a^ |  |
| +1 *SD* |  | **-0.07** | [-0.10, -0.05] | 0.02 | < .001 |  |  |  |  |  |
|  |  |  |  |  |  |  |  |  |  |  |
|  |  |  |  |  |  |  |  |  |  |  |
| Actor-partner |  | -0.10 | [-0.20, 0.01] | 0.05 | .065 |  | Power^a^ |  | Aggression^a^ |  |
|  |  |  |  |  |  |  |  |  |  |  |
| -1 *SD* |  | -0.03 | [-0.06, 0.00] | 0.02 | .097 |  |  |  |  |  |
| +1 *SD* |  | **-0.08** | [-0.12, -0.04] | 0.02 | < .001 |  |  | Psychopathy^p^ |  |  |
|  |  |  |  |  |  |  |  |  |  |  |
|  |  |  |  |  |  |  |  |  |  |  |
| Partner-partner |  | **-0.13** | [-0.21, -0.03] | 0.04 | .005 |  |  | Psychopathy^p^ |  |  |
|  |  |  |  |  |  |  |  |  |  |  |
| -1 *SD* |  | -0.02 | [-0.05, 0.01] | 0.01 | .164 |  | Power^p^ |  |  |  |
| +1 *SD* |  | **-0.07** | [-0.09, -0.05] | 0.01 | < .001 |  |  |  | Aggression^a^ |  |
|  |  |  |  |  |  |  |  |  |  |  |
|  |  |  |  |  |  |  |  |  |  |  |
| Partner-actor |  | -0.06 | [-0.14, 0.03] | 0.04 | .214 |  | Power^p^ |  |  |  |
|  |  |  |  |  |  |  |  |  | Aggression^a^ |  |
| -1 *SD* |  | **-0.03** | [-0.06, -0.01] | 0.01 | .027 |  |  |  |  |  |
| +1 *SD* |  | **-0.06** | [-0.09, -0.03] | 0.02 | .002 |  |  | Psychopathy^a^ |  |  |

*Notes.* ^a^ = actor variable. ^p^ = partner variable. Simple slope coefficients (values at -1 *SD*, +1 *SD*) are not standardized.
